# DCK is a promising prognostic biomarker and correlated with immune infiltrates in hepatocellular carcinoma

**DOI:** 10.1186/s12957-020-01953-1

**Published:** 2020-07-20

**Authors:** Danjun Song, Yining Wang, Kai Zhu, Lingyu Tian, Qiang Gao, Jian Zhou, Jia Fan, Xiaoying Wang

**Affiliations:** 1 Department of Liver Surgery, Liver Cancer Institute, Zhongshan Hospital, Fudan University, Key Laboratory of Carcinogenesis and Cancer Invasion of Ministry of Education, Shanghai, 200032 People’s Republic of China; 2 Institutes of Biomedical Sciences, Fudan University, Shanghai, People’s Republic of China

**Keywords:** Deoxycytidine kinase, Hepatocellular carcinoma, Poor prognosis, Immune infiltration

## Abstract

**Background:**

Deoxycytidine kinase (*DCK*), an enzyme in the nucleoside biosynthetic pathway, can affect the development of immune cells. However, the relationships between the expression of *DCK*, patient prognosis, and tumor-infiltrating immune cells (TIICs) in hepatocellular carcinoma (HCC) are still unclear.

**Methods:**

The expression of *DCK* in HCC was analyzed through the Oncomine and Tumor Immune Estimation Resource (TIMER) databases. The impact of *DCK* on clinical prognosis was investigated via the Kaplan-Meier plotter and verified in the Gene Expression Profiling Interactive Analysis (GEPIA) databases. The interrelationships between *DCK* expression and TIICs in HCC were analyzed by the TIMER database. Additionally, the relationship between *DCK* expression and immune cell gene markers was calculated through TIMER and GEPIA databases.

**Results:**

Compared with the adjacent normal tissues, high expression of *DCK* was observed in HCC tissues. Also, the higher expression of *DCK* was correlated to poorer prognosis in HCC patients, and it was associated with decreased survival in those with early stage and grade. Moreover, *DCK* expression was positively correlated with TIICs, including CD4^+^ and CD8^+^ T cells, B cells, monocytes, tumor-associated macrophages (TAMs), M1 and M2 macrophages, neutrophils, natural killer cells, and dendritic cells. Specifically, *DCK* expression levels were significantly associated with diverse immune gene marker sets, including those of Tregs and exhausted T cells.

**Conclusion:**

These findings suggest that *DCK* expression is correlated with patient outcomes and tumor infiltration cell levels in HCC patients. Additionally, the increased level of *DCK* was associated with marker genes of Tregs and exhaustion-related inhibitory receptors, suggesting the potential role of *DCK* in immunosuppression and immune escape. These findings suggest that *DCK* can function as a potential novel prognostic biomarker and reflect the immune infiltration status in HCC patients.

## Introduction

Hepatocellular carcinoma (HCC), one of the major primary hepatic tumors, is the fourth most common cause of cancer-related death worldwide [1]. HCC often develops from chronic liver inflammation, especially liver cirrhosis. The common risk factors for liver cancer are hepatitis B/C virus infection, alcohol consumption, and non-alcoholic fatty liver disease. Although a remarkable advancement has been achieved in diagnosis, surgical treatment, adjuvant therapy, and immune therapy [2, 3], the prognosis after resection is unsatisfactory due to the high rate of recurrence [4, 5].

Deoxycytidine kinase (*DCK*) is one of the essential enzymes of the nucleoside salvage pathway, and its expression is associated with the resistance to antiviral and anticancer chemotherapeutic agents [6–8]. *DCK* is highly expressed in lymphoid cells or tissues, such as thymus, spleen, lymph nodes, peripheral blood mononuclear cell, and bone marrow cells. Interestingly, *DCK* was expressed differently in breast and pancreatic cancers, and the expression levels were associated with patient prognosis in both types [9, 10]. A previous study analyzing eight microarray datasets comprising 521 human HCC tissues found that *DCK* was upregulated in HCC tissues [11]. However, prognostic values and molecular mechanisms of *DCK* in HCC are still unclear.

Tumor microenvironment and tumor-infiltrating immune cells (TIICs) are topics of interest and have shown an important role in cancer studies [12, 13]. Immune cells of tumor microenvironment play an important role in tumor progression in HCC. The immunosuppressive microenvironment of HCC contributes to immune tolerance and immune escape through different mechanisms [14]. *DCK* can have an impact on peripheral T cell homeostatic proliferation and survival, and its deficiency can influence lymphocyte development [15–17], indicating its potential role in the immune microenvironment. However, whether *DCK* could influence immune cell and tumor microenvironment contributing to tumor progression still need investigation.

In this study, we investigated the expression level of *DCK* and determined its correlation with cancer patient prognosis based on the online public databases such as the Oncomine, Kaplan-Meier plotter, Tumor Immune Estimation Resource (TIMER), and Gene Expression Profiling Interactive Analysis (GEPIA). Specifically, we analyzed the correlation between *DCK* expression and TIICs in HCC through the TIMER database. The findings in this study showed the important role of *DCK* in HCC and provided an interrelationship and an underlying mechanism between *DCK* and TIIC interactions.

## Materials and methods

### The expression of *DCK*

The *DCK* expression levels were analyzed in different cancer types using the Oncomine database (https://www.oncomine.org/resource/login.html) [18]. The parameters about the threshold were as follows: *p* value of 0.05, fold change of 1.5, and gene ranking of all. The results are exhibited as *p* value, fold changes, and rank (%).

### Prognosis analysis related to *DCK* expression in HCC patients

In order to determine the relationship between *DCK* expression and patient prognosis, Kaplan-Meier plots (http://kmplot.com/analysis/) were used in the HCC [19]. For the expression of the DCK, the expression between the lower and upper quartiles was analyzed and the best performing threshold was applied as the final cutoff value automatically in the Cox regression analysis. The results were presented with the hazard ratio (HR) and *p* values or Cox *p* values from a log-rank test.

### Immune infiltration analysis related to the *DCK* expression

The TIMER database (https://cistrome.shinyapps.io/timer/) [20] includes gene expression profiles and immune infiltration cells in 32 cancer types based on RNA-Seq expression profiling data from The Cancer Genome Atlas (TCGA) database. It can detect the differential gene expression in tumor tissues, analyze the infiltration of immune cells, and find the correlation between two genes through these profiles [21]. The infiltration levels of immune cells were analyzed through estimation by the statistical method through gene expression data. Therefore, we investigated the relationship between *DCK* expression and TIICs, including CD4^+^ and CD8^+^ T cells, B cells, neutrophils, dendritic cells, and macrophages. Additionally, the correlations among *DCK* expression and different gene markers of TIICs, like T cells, B cells, tumor-associated macrophages (TAMs), monocytes, M1 and M2 macrophages, natural killer (NK) cells, neutrophils, dendritic cells (DCs), T helper (Th) cells, T helper 17 (Th17) cells, follicular helper T (Tfh) cells, Tregs, and exhausted T cells, were analyzed. The marker genes of TIICs were reported as previous study [22].

### Gene correlation identification in GEPIA

The GEPIA database (http://gepia.cancer-pku.cn/index.html) contains the gene expression data from TCGA and the Genotype-Tissue Expression (GTEx) projects [23]. It can also analyze differential gene expression, patient prognosis, and relationship of two genes through online data. Therefore, gene expression levels related to patient prognosis were identified via the GEPIA database, and the interrelationships between the levels of gene expression and TIICs were established. A median value of the DCK expression was used as a cutoff to distinguish high expression from low expression of DCK.

### Statistical analysis

All statistical analyses were performed in R (version 3.5.2). Survival curves were generated by the Kaplan-Meier plots and GEPIA database. Forest plots were constructed using R package “forestplot” (https://cran.r-project.org/web/packages/forestplot/index.html). Spearman’s correlation was used to evaluate the relation between gene expression and infiltrating immune cells. The strength of the correlation was defined as follows: 0.00–0.29 (weak), 0.30–0.59 (moderate), 0.60–0.79 (strong), 0.80–1.00 (very strong) [24]. *p* values < 0.05 were considered as statistically significant except for the correlation analysis.

## Results

### The expression levels of *DCK* in HCC

**Fig. 1:**
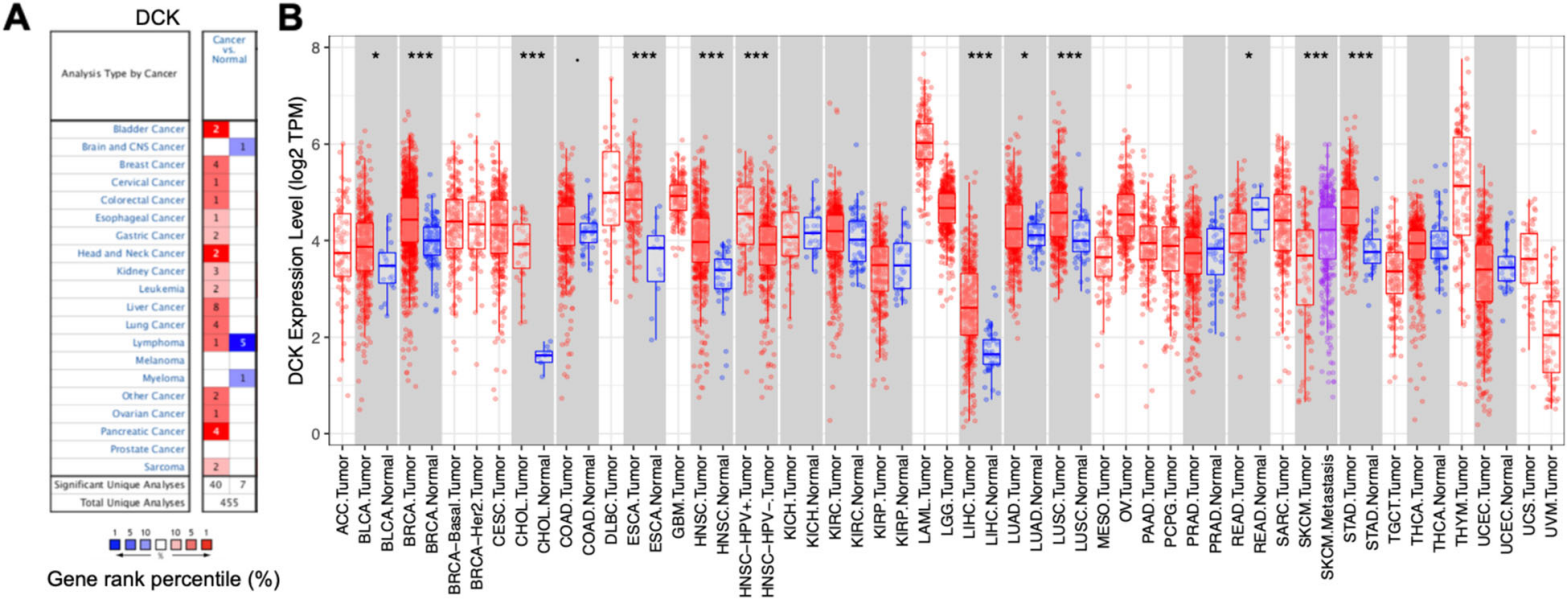
The expression of *DCK*. **a** High expression of *DCK* in HCC tissues compared with adjacent normal tissues by the Oncomine database. **b** The level of *DCK* expression in HCC from the TIMER database. *p < 0.05, **p < 0.01, ***p < 0.001

A previous study has reported that metabolic gene *DCK* was upregulated in the HCC tissues [11]. In order to validate the expression of *DCK* between HCC tissues and adjacent normal tissues, gene expression analysis was analyzed via the Oncomine database. We found that *DCK* expression was significantly higher in the HCC (Fig. 1a and Supplementary Table 1). Further evaluation of *DCK* expression in HCC was calculated using the TIMER database, and consistent results were found for HCC (Fig. 1b). These findings validated that *DCK* expression was highly expressed in HCC tissues.

### Prognostic values of *DCK* expression in HCC

**Fig. 2:**
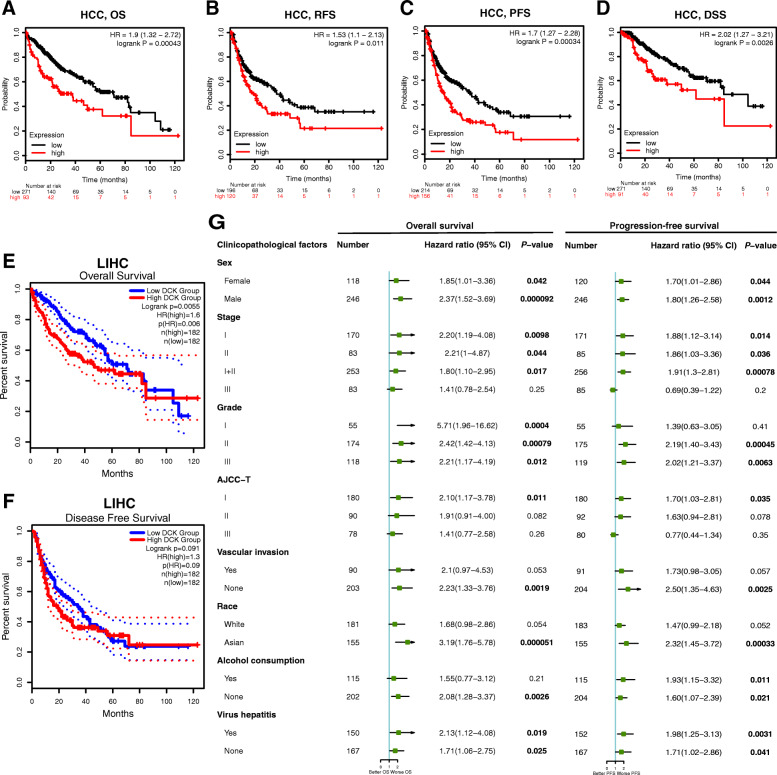
Prognostic values of *DCK* expression in HCC (**a**–**g**). **a**–**d** OS, RFS, PFS, and DSS in HCC cohorts through the Kaplan-Meier plots database (*n* = 364, *n* = 316, *n* = 370, *n* = 362, respectively). **e**–**f** OS and PFS in HCC cohorts through the GEPIA database (*n* = 364, *n* = 364, respectively). **g** Cox regression analysis of HCC patients in OS and PFS. OS overall survival, RFS relapse-free survival, PFS progression-free survival, DSS disease-specific survival

Next, we investigated the relationship between the *DCK* expression and prognosis in HCC using the Kaplan-Meier plotter database. Interestingly, a high expression of *DCK* was associated with poorer prognosis in HCC patients (overall survival (OS): HR = 1.90, 95% CI = 1.32–2.72, *p* = 0.00043, *n* = 364; relapse-free survival (RFS): HR = 1.53, 95% CI = 1.10–2.13, *p* = 0.011, *n* = 316; progression-free survival (PFS): HR = 1.70, 95% CI = 1.27–2.28, *p* = 0.00034, *n* = 370; disease-specific survival (DSS): HR = 2.02, 95% CI = 1.27–3.21, *p* = 0.0026, *n* = 362; Fig. 2a–d). These findings indicated the potential prognostic values of *DCK* in HCC. Also, we identified the relationship between the *DCK* expression and prognostic values in HCC using the GEPIA database. The results showed that high expression of *DCK* was associated with dismal prognosis in OS of HCC patients (Fig. 2e), but there was not statistical difference in PFS (Fig. 2f). These findings suggest that the expression of *DCK* influences the prognosis of HCC patients.

### High expression of *DCK* related to clinical characteristics of HCC patients

Based on the differential expression of *DCK* and significant prognostic values related to its expression, which were observed in HCC, we investigated the relationship between the expression of *DCK* and different clinicopathological characteristics of HCC using the Kaplan-Meier plotter database. The high expression of DCK was related to worse OS and PFS rates in the females (OS: HR = 1.85, *p* = 0.042; PFS: HR = 1.70, *p* = 0.0013) and males (OS: HR = 2.37, *p* = 9.20 × 10^− 5^, PFS: HR = 1.80, *p* = 0.0012), Asians (OS: HR = 3.19, *p* = 5.10 × 10^− 5^; PFS: HR = 2.32, *p* = 0.00033), non-alcoholics (OS: HR = 2.08, *p* = 0.0026; PFS: HR = 1.60, *p* = 0.021), patients with hepatitis viral infection (OS: HR = 2.13, *p* = 0.019; PFS: HR = 1.98, *p* = 0.0031) and those without it (OS: HR = 1.71, *p* = 0.025; PFS: HR = 1.71, *p* = 0.041), and patients without vascular invasion (OS: HR = 2.23, *p* = 0.0019; PFS: HR = 2.50, *p* = 0.025) (Fig. 2g). Specifically, high expression of *DCK* was correlated with poorer OS and PFS rates in stages I, II, and I+II, grades II and III, and AJCC-T stage I patients, but was not associated with stage III and AJCC-T II and III stages (Fig. 2g). These findings indicate that the expression level of *DCK* can influence the prognosis in HCC patients with different clinicopathological factors, especially in these early-stage patients.

### *DCK* expression levels are correlated with the immune infiltration in HCC

**Fig. 3:**
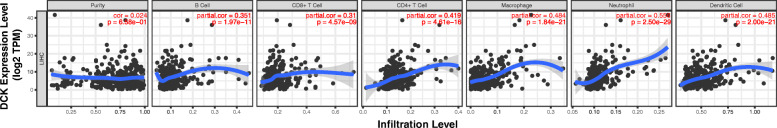
Relationship between *DCK* expression and infiltration levels of immune cells in HCC via the TIMER database. The expression of *DCK* was significantly correlated with the infiltration levels of B cells, CD8^+^ T cells, CD4^+^ T cells, macrophages, neutrophils and dendritic cells in HCC

Tumor-infiltrating lymphocytes influence the survival of patients with cancer. Therefore, the interrelationship between the TIICs and the *DCK* expression was investigated by the TIMER database. The high expression of *DCK* was related to patient outcomes in HCC, and it was correlated with high infiltration levels of immune cells (Fig. 3). The *DCK* expression was associated with dismal outcomes and positively correlated with the infiltration levels of B cells (cor = 0.351, *p* = 1.97 × 10^− 11^), CD8^+^ T cells (cor = 0.310, *p* = 4.57 × 10^− 9^), CD4^+^ T cells (cor = 0.419, *p* = 4.61 × 10^− 16^), macrophages (cor = 0.484, *p* = 1.84 × 10^− 21^), neutrophils (cor = 0.556, *p* = 2.50 × 10^− 29^), and dendritic cells (cor = 0.485, *p* = 2.00 × 10^− 21^) in HCC (Fig. 3). These findings suggest immune infiltration may play a role in patient outcomes, and *DCK* could modulate immune infiltrating cells into HCC tissues. However, the expression of *DCK* was not correlated with tumor purity (cor = 0.024, *p* = 6.58 × 10^− 1^), suggesting the expression of *DCK* was from cells in the tumor microenvironment.

### Correlation between *DCK* expression and different gene markers of immune cell subsets

We further investigated the relationships between *DCK* expression and multiple TIICs in HCC through TIMER and GEPIA databases based on the different immune cell gene markers. The correlation was adjusted for tumor purity due to its influences on the immune infiltration analysis. The immune cells included CD4^+^ T cells, CD8^+^ T cells, B cells, TAMs, monocytes, M1 and M2 macrophages, neutrophils, dendritic cells, and NK cells in HCC patients. Also, subsets of T cells, including Th1, Th2, Tfh, Th17, Tregs, and exhausted T cells, were investigated.

**Fig. 4:**
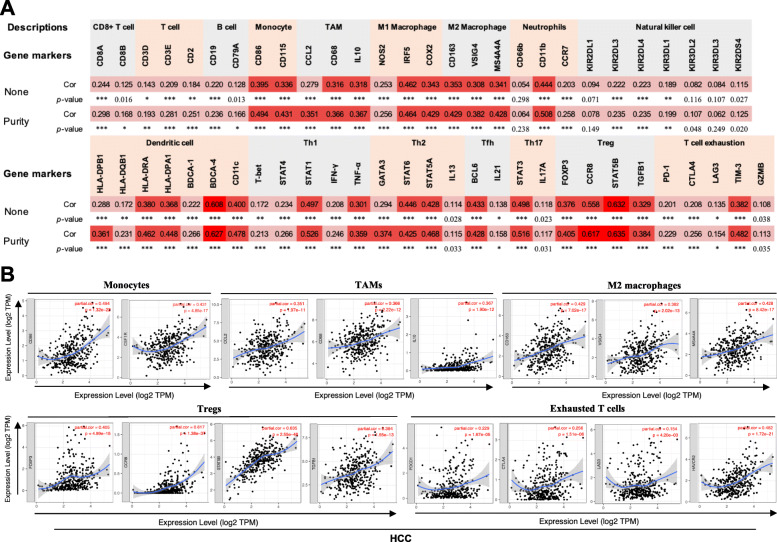
Correlation between the expression of *DCK* and marker genes of infiltrating immune cells in HCC using the TIMER database. **a** Correlation between the expression of *DCK* and immune molecular genes. None, correlation without adjustment; Purity, correlation adjusted by purity; 0≤***<0.0001≤**<0.001≤*<0.01. **b** The scatter plots of correlation between *DCK* expression and the gene markers of monocytes, TAMs, M2 macrophages, Tregs and exhausted T cells in HCC. TAMs tumor-associated macrophage

We found that the *DCK* expression was significantly correlated with the expression of gene markers in monocytes, TAM, and M2 macrophages in HCC patients after adjusting tumor purity (Fig. 4a, b). *DCK* expression was significantly associated with subsets of T cells, revealing its association with cytokine secretion (Fig. 4a, b). Tregs have an essential role in immune escape and angiogenesis, and immune checkpoint regulation is important for T cell-mediated cancer-killing effect. Interestingly, the expression of *DCK* was associated with the gene markers like *CCR8*, *STAT5b*, and *TGFB1* of Tregs, and *PD–1*, *CTLA–4*, *LAG3*, *TIM–3*, and *GZMB* of exhausted T cells after adjusting tumor purity (Fig. 4a, b), revealing that increased expression of *DCK* was associated with immunosuppression in HCC. These findings suggest that immune infiltrating cells could influence patient prognosis in HCC. Similar expression results were observed in GEPIA (Supplementary Table 2). The results strongly indicated the potential functions of *DCK* in contributing to the angiogenesis and regulating immune escape in HCC.

## Discussion

In this study, we demonstrate the *DCK* expression and corresponding patient prognosis in HCC. *DCK* expression was significantly higher in HCC patients, and its high expression was correlated with worse long-term outcomes. To the best of our knowledge, this is the first study that reported a prognostic impact of *DCK* in HCC. Moreover, high expression of *DCK* was correlated with unfavorable prognosis for HCC patients, especially for those with early stage. Additionally, the expression of *DCK* was correlated with TIICs and its marker genes in HCC. The increased level of *DCK* was correlated with marker genes of Tregs and exhaustion-related inhibitory receptors, suggesting potential mechanisms of *DCK* in immune escape. These findings demonstrate that *DCK* can be a promising prognostic biomarker and is correlated with immune infiltrates in HCC.

As an essential enzyme in the nucleoside biosynthetic pathway, *DCK* plays a role in metabolism and DNA synthesis during the embryogenesis, organogenesis, and essential cell developmental processes. The deficiency of *DCK* in mice blocks the lymphocyte development and shows a significant decrease in both T and B cell populations compared with wild-type mice [17]. Mutation of *DCK* impairs T and B cell function in mice, and mutant contains a CD44^high^CD62L^low^ memory phenotype lymphocytes in the peripheral, with high levels of proliferation and apoptosis [15]. However, the activation of *DCK* elevates the level of deoxyadenosine triphosphate (dATP) intracellularly, which suggests the link of *DCK* activation to the induction of apoptosis. Therefore, differentially expressed *DCK* in tumor tissues could influence the immune cell functions.

There is an urgent need for improving early diagnosis and treatment of HCC patients. The development of new biomarkers in detection, prognostic assessment, and treatment options should be a major research interest in the future [25]. The high expression of *DCK* was observed in liver tumors, and its low expression was found in normal liver. The high expression of *DCK* was correlated with dismal outcomes. Therefore, we have reason to believe that the *DCK* could be a prognostic biomarker in HCC. Tumor microenvironment are important for tumor progression. Recent studies demonstrated that the integration of TIICs and clinicopathologic characteristics can be a prognostic predictive model and predict the response of immune therapy [13, 26]. A positive correlation between the *DCK* expression and the TIICs indicates that *DCK*-based prediction of patient outcome in HCC may be associated with immune cell infiltration. Negative association between *DCK* and tumor purity suggested that *DCK* expression was from cells in the tumor environment. The expression of *DCK* was more likely from TIICs [27]. Moreover, the expression of *DCK* was associated with the expression of the immune cell markers, such as the markers of monocytes, TAM, M1, and M2 macrophages. These findings suggest the potential functions of *DCK* in regulating infiltration and activity of macrophages. Furthermore, we observed that *DCK* expression was relevant to the expression of markers of T cell subsets, like Th1, Th2, Tfh, and Th17, indicating its potential influence on the tumor progression in regulating the secretion of cytokines from the helper T cells.

Interestingly, the expression of *DCK* was correlated with the marker genes of Tregs and exhaustion-related inhibitory receptors. Tregs play an important part in immune escape and angiogenesis, which could influence patient outcomes. Overexpression of inhibitory receptors in HCC can inhibit immune response and dampen T cell functions, resulting in tumor progression [28–31]. In return, the anti-immune-checkpoint inhibitor can provide HCC patients with a survival benefit [32]. These findings suggest the role of *DCK* as an essential aspect in the immunosuppressive and immune escape and also indicate its potential function in regulating TIICs in HCC patients. Moreover, cytosolic Ca^2+^ ions are associated with the activity of *DCK* in cells [33]. Altered Ca^2+^ flux could promote T cell subsets, which in turn promote cytokine production and downregulate *CTLA–4* and *PD–1* expression [34]. In addition, altered Ca^2+^ influences Ca^2+^/cyclic AMP (cAMP) signaling pathway contributing to the regulation of cytokines [35]. Both the excretion of cytokines and change in cAMP pathway are associated with tumor progression [12, 36]. These findings could be the potential mechanism of *DCK* regulating the expression of inhibitor receptors and contributing to the progression of a tumor.

This study had several limitations. First, the role of *DCK* was investigated through various datasets. Some potential basis still exists because of the different methods in data collection and analysis. Second, the correlation coefficients were not very strong. In fact, when analyzing the relationship between gene levels and TIICs, the correlation coefficients were mostly uncorrelated or had a weak and moderate correlation, and strong correlations were rare [22, 24]. Third, we only used public datasets, further identified by our HCC patient profiles, and the detailed relationship should be confirmed by in vitro or animal experiments. Additionally, comparing the efficiency between *DCK* and traditional biomarkers in HCC should be further studied. The function of *DCK* in other cancer types should be investigated in the future.

## Conclusions

In conclusion, our results suggested that elevated *DCK* expression levels were associated with dismal prognosis together with enhanced immune infiltration in HCC, and the increased level of *DCK* was correlated with marker genes of Tregs and exhaustion-related inhibitory receptors, which could be the potential mechanism of *DCK* affecting patient outcomes. Therefore, relatively high levels of *DCK* in HCC could indicate the increased immune infiltration status and reflect a higher risk of death.

## Supplementary information

**Additional file 1: Table S1.** The expression of *DCK* in hepatocellular carcinoma *versus* normal tissues in the Oncomine database. **Table S2.** Correlation analysis between *DCK* and related genes and markers of immunes cells in GEPIA.

## Data Availability

All datasets used and/or analyzed during the current study are available from the public database.

## Abbreviations

DCK: Deoxycytidine kinase
TIICs: Tumor-infiltrating immune cells
TIMER: Tumor Immune Estimation Resource
GEPIA: The Gene Expression Profiling Interactive Analysis
HCC: Hepatocellular carcinoma
TAMs: Tumor-associated macrophages
TCGA: The Cancer Genome Atlas
NK cells: Natural killer cells
DCs: Dendritic cells
Th cells: T helper cells
Th17 cells: T helper 17 cells
Tfh cells: Follicular helper T cells
GTEx: The Genotype-Tissue Expression
OS: Overall survival
RFS: Relapse-free survival
PFS: Progression-free survival
DSS: Disease-specific survival
cAMP: Cyclic AMP
