# Supplementary material for: DCK is a promising prognostic biomarker and correlated with immune infiltrates in hepatocellular carcinoma
Source: World J Surg Oncol. 2020 Jul 20;18:176. doi: 10.1186/s12957-020-01953-1 (PMC7372783; doi:10.1186/s12957-020-01953-1)
Supplement: Supplementary file 1 — Additional file 1: Table S1. The expression of DCK in hepatocellular carcinoma versus normal tissues in the Oncomine database. Table S2. Correlation analysis between DCK and related genes and markers of immunes cells in GEPIA. [file 12957_2020_1953_MOESM1_ESM.docx]

**Supplementary Table 1. The expression of *DCK* in hepatocellular carcinoma *versus* normal tissues in the Oncomine database.**

| **Cancer type** | **P-value** | **Fold change** | **Rank (%)** | **Samples** | **Reference (PMID)** |
| --- | --- | --- | --- | --- | --- |
| Hepatocellular Carcinoma | 1.76E-10 | 2.255 | 2% | 57 | 19098997 |
| Hepatocellular Carcinoma | 8.67E-6 | 1.949 | 4% | 45 | 17393520 |
| Hepatocellular Carcinoma | 2.36E-7 | 2.186 | 4% | 43 | 21159642 |
| Hepatocellular Carcinoma | 9.18E-47 | 1.942 | 5% | 445 | 21159642 |
| Hepatocellular Carcinoma | 4.54E-9 | 1.532 | 7% | 180 | 12058060 |

**Supplementary Table 2. Correlation analysis between *DCK* and related genes and markers of immunes cells in GEPIA.**

| **Description** | **Gene markers** | **HCC** | |
| --- | --- | --- | --- |
|  |  | **cor** | ***p*** |
| **Monocyte** | *CD86* | 0.41 | *** |
|  | *CD115* | 0.38 | *** |
| **TAM** | *CCL2* | 0.27 | *** |
|  | *CD68* | 0.34 | *** |
|  | *IL10* | 0.34 | *** |
| **M2 Macrophage** | *CD163* | 0.15 | * |
|  | *VSIG4* | 0.29 | *** |
|  | *MS4A4A* | 0.33 | *** |
| **Tregs** | *FOXP3* | 0.26 | *** |
|  | *CCR8* | 0.54 | *** |
|  | *STAT5B* | 0.62 | *** |
|  | *TGFB1* | 0.25 | *** |
| **T cell exhaustion** | *PD-1* | 0.21 | *** |
|  | *CTLA4* | 0.25 | *** |
|  | *LAG3* | 0.049 | 0.35 |
|  | *TIM-3* | 0.38 | *** |
|  | *GZMB* | 0.036 | 0.5 |

HCC, hepatocellular carcinoma; TAM, tumor-associated macrophage; Cor, R value of Spearman’s correlation; None, correlation without adjustment. Purity, correlation adjusted by purity. **p* < 0.01; ***p* < 0.001; ****p* < 0.0001.
